# Combined Effects of Withaferin A and Sodium Butyrate on NF-κB Signaling and Epigenetic Regulation in Breast Cancer Cells

**DOI:** 10.3390/nu18061015

**Published:** 2026-03-23

**Authors:** Brittany L. Witt, Neha Singaravelan, Trygve O. Tollefsbol

**Affiliations:** 1 Department of Biology, College of Arts and Sciences, University of Alabama at Birmingham, Birmingham, AL 35233, USA; bwitt95@uab.edu (B.L.W.); nsingara@uab.edu (N.S.); 2 O’Neal Comprehensive Cancer Center, Heersink School of Medicine, University of Alabama at Birmingham, Birmingham, AL 35294, USA; 3 Integrative Center of Aging Research, University of Alabama at Birmingham, Birmingham, AL 35294, USA; 4 Nutrition Obesity Research Center, University of Alabama at Birmingham, Birmingham, AL 35294, USA; 5 Comprehensive Diabetes Center, Heersink School of Medicine, University of Alabama at Birmingham, Birmingham, AL 35294, USA

**Keywords:** breast cancer, NF-κB, withaferin A, sodium butyrate, epigenetic regulation, DNA methyltransferases, histone deacetylase

## Abstract

**Background/Objectives**: There is a clear need for more options to control the progression of breast cancer and prevent the occurrence of breast cancer in minority populations that have a higher rate of mortality due to triple-negative breast cancer (TNBC) subtypes. Prevalent nutraceuticals such as Ashwagandha (also known as the Indian Winter Cherry) have anti-inflammatory and apoptotic capabilities, as well as the ability to inhibit cancer growth. The purpose of this study is to analyze the novel combination of withaferin A (derived from the Indian Winter Cherry and known to have histone deacetylase inhibition capabilities) and sodium butyrate (a short-chain fatty acid produced from the gut microbiome and known to have DNA methyltransferase inhibition capabilities) treatment on breast cancer-derived cell lines. There is a scientific gap of possible causality of decreasing breast cancer progression when treated with sodium butyrate and withaferin A. **Methods**: Two in vitro cell viability assays were utilized consisting of [MTT (4,5 Dimethylthiazol-2-yl)] and the neutral red assay to analyze the impact of treatment of compounds alone and in combination on breast cancer cells for 72 h. The Highest Single Agent (HSA) combination analysis was utilized to derive combination indexes for our breast cancer cell types. Protein and gene expression was investigated for Class 1 histone deacetylases, de novo DNA methyltransferase, the p65 subunit of NF-κB, and *NFκB1*. Lastly, DNA methyltransferase enzymatic activity was analyzed via the Epigentek DNMT Activity/Inhibition ELISA Easy Kit. **Results**: Through the cell viability assay [MTT (4,5 Dimethylthiazol-2-yl)], MCF−7, MDA−MB−231, and MDA−MB−157 cells were found to have a decrease in cell viability due to combinatorial treatment with withaferin A and sodium butyrate. Western blot results depicted a decrease in protein expression levels for DNA methyltransferases due to the administration of 2.5 mM sodium butyrate and 0.2 µM withaferin A alone and in combination for breast cancer cell lines MCF−7, MDA-MB-231, and MDA−MB−157. Additionally, the combination of these two components have successfully inhibited the progression of the *NFκB1* gene within analysis through the quantitative polymerase chain reaction (qPCR). **Conclusions**: The novel combination of withaferin A and sodium butyrate have markedly reduced the progression of breast cancer-derived cell lines for cell viability, epigenetic *DNMT* gene expression, as well as inhibiting *NFκB1* signaling on the gene expression level.

## 1. Introduction

Breast cancer is the most common cancer in American women, outside of skin cancer; and 1 in 8 or 13% of the female population in the U.S. will develop breast cancer in their lifetime. Black women are 40% more likely to die from breast cancer than white women, and some studies state it may be due to the higher chance of them acquiring the triple-negative molecular subtype [1]. This disease is known to be a malignant growth of tissue within the breast that could metastasize to various parts of the body. Many scientists have studied diverse ways to decrease the progression and chance of the disease from occurring later in life by monitoring physical activity, diet, and reducing the amount of toxic substances within the environment. While these strategies have been shown to have some clinical evidence to prevent breast cancer, there are still several gaps and limitations. For example, the evidence of the effect of physical activity to reduce breast cancer is weak because a large part of data are cohort or case-control studies rather than randomized controlled trials. Furthermore, the specific biological explanation for the protective effect of exercise in breast cancer is uncertain since the biochemical pathways that are affected cannot yet be conclusively described [2].

There are increased studies of the benefits of a good diet to prevent breast cancer. Diets rich in fruits, vegetables, and whole grains are associated with a lower risk of developing breast cancer as they regulate metabolic and hormonal factors that are linked to cancer development. Specific plant-based diets can reduce oxidative stress and chronic inflammation [3]. Certain dietary components can also reduce estrogen levels and since breast cancers are hormone dependent, blocking estrogen signaling can reduce risk [4].

Polyphenols are naturally occurring compounds that are found in fruits, vegetables, and beverages. They are secondary metabolites of plants and are used in defense against ultraviolet radiation or pathogens [5]. Besides their plant specific roles, they can also act as powerful antioxidants which allows them to neutralize reactive oxygen species that damage DNA and can initiate cancer. They are also able to interfere with cell signaling pathways that control cell division and activate apoptosis [6]. Specifically for breast cancer, polyphenols can bind to estrogen receptors, inhibit aromatase activity, and modulate specific breast cancer signaling pathways such as the NF-κB pathway and ERK signaling pathway [7].

The gut microbiome has molecules that can be used for breast cancer prevention. Short-chain fatty acids such as butyrate, propionate, and acetate can inhibit tumor cell proliferation and reduce inflammation. Secondary bile acids also have the ability to reduce breast cancer cell proliferation by inhibiting the epithelial-to-mesenchymal transition. The gut microbiome also has bacteria that can convert plant compounds into estrogen-like molecules that compete with estrogen for receptors and act as anti-estrogen agents [8,9].

Although there have been many studies that involve modifying the diet and capitalizing on the gut microbiome as a breast cancer prevention, to our knowledge, there are very few studies that evaluate the consumption of a product from the gut microbiome that has epigenetic mechanisms in combination with another dietary component known to decrease stress and anxiety. There is a need in the field to elucidate the connection between the gut microbiome and breast cancer growth involving substances that have epigenetic mechanisms.

Sodium butyrate is a short-chain fatty acid produced by gut microbiota. It is a known histone deacetylase (HDACs) inhibitor, particularly for class 1 and 2 HDACs, inducing changes in gene expression. Butyrate has the ability to contribute to many anti-inflammatory functions, including cytokine modulation, inhibition of NF-kB pathway, reduction of nitric oxide, and activation of Aryl Hydrocarbon receptor. Specifically, sodium butyrate has been shown to have anti-tumorigenic mechanisms as it can induce cell cycle arrest via p21/Cip1, inhibit stem cell proliferation, and change Wnt and PPARy pathways [10]. One study examined the effects of sodium butyrate on transformed fibroblast cells, specifically E1A + Ras mouse embryonic fibroblasts (MEFs) (oncogene-driven), versus normal fibroblast cells. Results showed that sodium butyrate inhibited double-strand break repair and exogenous DNA repair in transformed cells only. In addition, sodium butyrate was able to alter DNA damage response protein levels and complex stability, including downregulation of Ku80, MRN complex destabilization, and increased hypophosphorylation of Mre11 in the transformed but not the normal cells [11]. Another study examined the effect of sodium butyrate’s impact on Toll-like receptor 4 (TLR4) in cancer. Results showed that sodium butyrate reduced cell viability in the prostate cancer cell line, increased late apoptotic cell population, and decreased TLR3 signaling, suppressing pro-inflammatory mechanisms [12].

Withaferin A (WA) is a natural compound derived from *Withania somnifera* and more commonly known as Ashwagandha or Indian Winter Cherry. It has the ability to regulate cholesterol homeostasis, suppress NF-κB-driven inflammatory and angiogenic gene expression, and modulate antioxidant mechanisms [13]. WA has specific anticancer mechanisms as it can inhibit DNA methyltransferases (DNMT) and suppress tumor progression [14]. One study explored Withaferin A’s effects on triple-negative breast cancer (TNBC). Results showed that WA induced gene-specific methylation in tumor-promoting genes in MDA-MB-231 cells but no methylation in the MCF-7 control cell line. In addition, WA hypermethylation had transcriptional repression in genes involved in metastasis and cell cycle progression [15]. Another study investigated WA effects on melanoma cells. Cell viability assays showed that WA selectively kills melanoma cells, inhibits melanoma cell migration, and induces necrosis through TRIM16 [16].

While there have been many studies that have explored the individual uses of WA and sodium butyrate to suppress cancer growth, there are limited studies that explore these polyphenols in combination. Thus, this study strives to explore the combinatorial effect of withaferin A and sodium butyrate in breast cancer cell lines. Even though breast cancer cells and gastrointestinal cells are different, the possibility of metastasis from breast to the GI can be conducted through similar pathways, including the NF-κB pathway via inflammation.

## 2. Materials and Methods

### 2.1. Cell Lines

ER (+) MCF−7 and triple-negative [ER (−) PR (−)] MDA-MB-157 breast cancer cell lines were used in this study. MCF−7 cells were derived in the 1970s from a 69-year-old white female patient with metastatic pleural effusion. MDA−MB−157 cells were derived in 1972 from a 44-year-old black female patient with metastatic breast cancer and pleural effusion. MDA−MB−231 cells were derived from a 51-year-old white female by isolating the mammary gland of an adenocarcinoma via metastatic pleural effusion in 1973. By utilizing both hormone-dependent and independent breast cancer cell lines, we were able to investigate the efficacy of nutraceuticals in various environments. A noncancerous control cell line, MCF10A, was also utilized. All cell lines were obtained from ATCC (American Type Culture Collection, Manassas, VA, USA).

### 2.2. Chemicals and Treatments

Sodium butyrate (+98% pure), C_4_H_7_NaO_2_, has a molecular weight of 110.09 g/mol and was obtained from ThermoFisher Scientific (Waltham, MA, USA). Withaferin A (WA), C_28_H_38_O_6_, has a molecular weight of 470.606 g/mol and was acquired from Sigma-Aldrich (St. Louis, MO, USA). Sodium butyrate (NaB) was dissolved in deionized water with a 100 mg/mL stock concentration and stored at −20 °C. WA was diluted in dimethyl sulfoxide (DMSO), also purchased from Sigma-Aldrich, and stored in stocks of 10 mmol/L at −20 °C.

Cells were seeded and allowed to adhere for 24 h, after which they were treated for a three-day period with individual and combinatorial dosages of WA and NaB. The control vehicle for each grouping was DMSO. The treatment solutions were freshly prepared for each cultured cell investigation, and the cultured media was fresh when replaced every 24 h.

### 2.3. Cell Culturing

MCF−7, MDA−MB−231, and MDA−MB−157 breast cancer cells were subcultured in Dulbecco’s Modified Eagle’s Medium DMEM 1X (Thermo Fisher Scientific, Waltham, MA, USA) and supplemented with 10% fetal bovine serum (GeminiBio, West Sacramento, CA, USA) and 1% penicillin/streptomycin (Millipore, Visalia, CA, USA). The MCF10A control cells were grown in DMEM F12 media (Thermo Fisher Scientific, Waltham, MA, USA) accompanied with 5 mL penicillin/streptomycin, 12.5 µL of hydrocortisone, 500 µL of insulin, 100 µL of EGF, 100 µL of 1 mg/mL cholera toxin, and 25 mL of fetal bovine serum. All cells were subcultured according to ATCC protocols and maintained under humid conditions of 5% CO_2_ and 95% air at 37 °C.

### 2.4. Cell Viability Assays

The 3-(4,5-dimethylthiazol-2-yl)-2,5-diphenyltetrazolium bromide (MTT) assay and neutral red assay were performed to analyze the cellular viability of breast cancer cells and control cells after treatment with various concentrations of NaB or WA, along with selected concentrations of the combined drugs. Cells were seeded in triplicate and allowed to attach to a 96-well plate for 24 h. They were then treated with the NaB and/or WA over a 72 h period. On the fourth day, 20 µL of MTT reagent (Sigma, St. Louis, MO, USA) dissolved from 5 g/L in phosphate-buffered saline (PBS) was added into each well and allowed to incubate for 3 h at 37 °C. After incubation, MTT reagent was removed and 200 µL of DMSO was added to each well and incubated for 15 min wrapped in aluminum foil. The absorbance readings were obtained at 570 nm using a spectrophotometer (BioRad, Hercules, CA, USA).

The neutral-red assay was completed by use of the manufacture’s guide provided by a neutral assay kit (Abcam, Fremont, CA, USA). After treatment of cells similar to MTT for 72 h, culture media was removed, and cells were fixed with 4% paraformaldehyde. Following washing, 150 µL neutral red staining solution was added to each well, and the plate was incubated at 37 °C for 2 h. After incubation, the cells were gently washed again with washing solution and 150 µL of 1X Solubilization Solution was added to each well and incubated for 20 min at room temperature. The absorbance at 540 nm was read using a spectrophotometer (BioRad, Hercules, CA, USA).

### 2.5. Western Blotting Analysis

After 72 h treatment of cell culture lines with individual and combinatorial dosages of WA and/or NaB, cell lysates were obtained by resuspension within 1 mL of RIPA lysis buffer (Upstate Biotechnology, Charlottesville, VA, USA) and 10 µL of 100X Phosphatase/Protease inhibitor (Cell Signaling Technology, Danvers, MA, USA). The Bradford Assay was then performed to evaluate the allotted protein concentration for the extracts. 15 to 20 µL of equal concentration protein lysates were loaded into 4–15% NuPAGE Tris-HCl precast gels (Invitrogen, Carlsband, CA, USA). The separated proteins were transferred onto a nitrocellulose membrane using the Trans-Blot Turbo Transfer System (Bio-rad, Hercules, CA, USA). Probing of the nitrocellulose membrane was executed with the following primary antibodies: HDAC1, HDAC2, HDAC3, HDAC8, DNMT3A, and DNMT3B. All antibodies were obtained from Cell Signaling Technology along with β-actin, which was an internal control for each membrane. Following the incubation with a secondary antibody, immunoreactive bands were visualized using Clarity MaxTM Western ECL Blotting Substrates (Bio-rad, Hercules, CA, USA). Images were visualized using ChemiDocTM Imaging Systems (Bio-rad, Hercules, CA, USA) and quantification of proteins was conducted with the utilization of Image J software (v1.53e) in combination with statistical analysis via GraphPad Prism (version 11, 84th edition).

### 2.6. Quantitative Real-Time PCR

Total RNAs from cells were extracted utilizing the RNeasy kit from Qiagen (Valencia, CA, USA) according to the manufacturer’s instructions. The iScript cDNA synthesis kit (Bio-Rad) was further used to reverse transcribe the isolated RNA. Gene target expressions were performed in triplicate on three randomly selected independent samples from each group using SsoAdvanced Universal SYBR Green Supermix (Biorad, Hercules, CA, USA). Specific gene primers for *DNMT3A*, *DNMT3B*, *HDAC1*, *HDAC2*, *HDAC3*, *HDAC8*, *NFκB1*, and *GAPDH* (Supplementary Table S2) were synthesized and purchased from Integrated DNA Technologies (Coralville, IA, USA). For the PCR analysis, 1 µL of cDNA was used with 7 µL of nuclease-free water, 10 µL of SYBER green, and 2 µL of reverse and forward primers. The real-time PCR was performed using CFX Connect Real-Time system (Bio-Rad) with GAPDH (Glyceraldehyde 3-phosphate dehydrogenase) in parallel as a control. Thermal cycling was initiated for 3 min at 95 °C followed by 39 PCR cycles (95 °C for 10 s, 55 °C for 30 s, 72 °C for 30 s).

### 2.7. DNA Methyltransferase (DNMT) Activity Assays

Nuclear extracts were prepared using the EpiQuik nuclear extraction kit from EpiGenTek (OP-0002-1) (Farmingdale, NY, USA) and the manufacturer’s procedure was followed. The nuclear extracts were then used for determination of overall DNMT enzymatic activities using the EpiQuick DNMT Activity/Inhibition ELISA Easy Kit (Colormetric) (Epigentek, Farmingdale, NY, USA).

### 2.8. Statistical Analysis

Error bars represent the standard error of the mean (SEM). Each experiment described was completed in triplicate with 3 to 6 technical replicates. *p* < 0.05, <0.01, <0.001, and <0.0001 are significant. All comparisons were made with the control. Also, statistical significance between treatment and control groups was evaluated by one-way independent ANOVA followed by Dunnett’s test for multiple comparison by using Graphpad Prism software (version 10.4.1). All graphs were created by GraphPad Prism (version 11, 84^th^ edition) or BioRender (BioRender.com).

## 3. Results

### 3.1. Combinatorial NaB and WA Synergistically Decreased the Cellular Viability of Breast Cancer Cells

**Figure 1:**
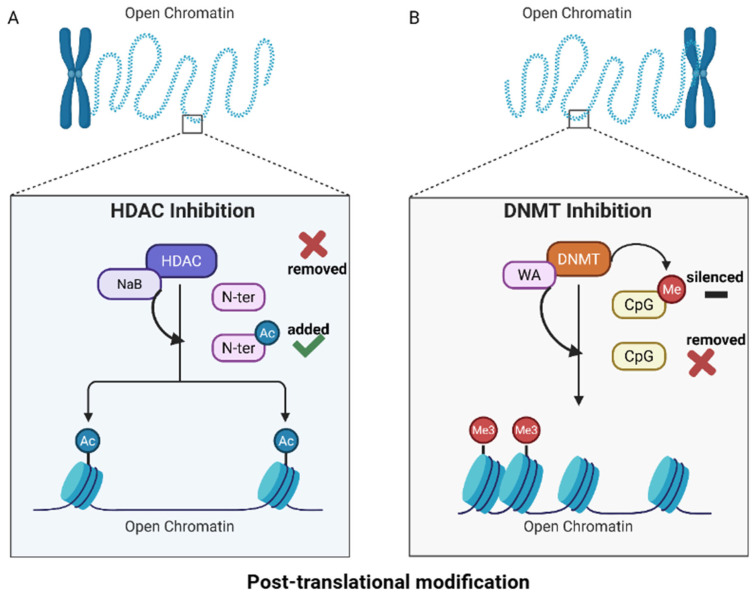
Epigenetics and gene expression schematic. (**A**) Bioactive dietary components such as NaB and sulforaphane (SFN) have HDAC inhibition capabilities, resulting in the addition of an acetyl-group (Ac) to the N-terminal (N-ter) tails within chromatin to open the structure. (**B**) Withaferin A and epigallocatechin−3−gallate (EGCG) have DNMT inhibition capabilities that result in the loss of the methyl groups (Me) located on cytosine-phosphate-guanine (CpG) islands that silence specific genes within DNA. Once these methyl groups are removed, the resulting post-translational modifications can lead to activation of previously silenced genes. Abbreviations: HDAC; histone deacetylase—enzyme, NaB; sodium butyrate—short chain fatty acid, N-ter; n-terminal tail, Ac; acety-group, Me; methyl groups, DNMT; DNA methyltransferase—enzyme, WA; withaferin A—steroidal lactone, CpG; cytosine-phosphate-guanine islands. Created in BioRender. Lab, T. (2026) https://BioRender.com/8n2szz8. (accessed on 13 March 2026).

We have investigated the combination and single dose usage administrative effects of short-chain fatty acid sodium butyrate (NaB) and phytochemical withaferin A (WA) on breast cancer cell lines MDA−MB−231, MDA−MB−157, and MCF−7. Both substances have been utilized previously within our laboratory to show a decrease in histone deacetylase (HDAC) activity and DNA methyltransferase (DNMT) activity for NaB and WA, respectively [14,17]—the molecular mechanism for the action of these two anti-inflammatory agents are illustrated by Figure 1.

This schematic of the dietary agents’ ability to inhibit HDAC and DNMT mechanisms is a key aspect that impacts epigenetics regulation [18]. The diet’s ability, environmental factors, exposure to harmful substances, and stress can change gene expression but not alter the DNA sequence. Another aspect of epigenetic regulation involves RNA-based processes, and resveratrol, a naturally occurring phytochemical found in grapes, has been shown to reduce mRNA stability without altering the promoter activity [19]. Figure 1A depicts the ability of NaB and sulforaphane (SFN) to impede the HDAC enzyme’s ability to remove acetyl groups during post-translational modification. These HDAC inhibition abilities cause the chromatin structure to be open and readily available for gene expression. Figure 1B illustrates the capabilities of dietary agents such as WA and EGCG to inhibit the DNMT enzyme from adding a methyl group on CpG islands that silence genes.

**Figure 2:**
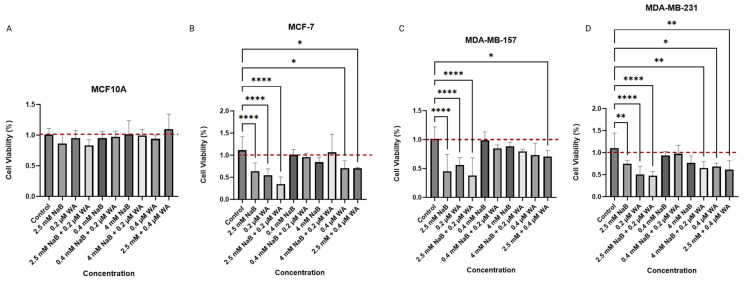
Inhibition of cellular viability by various dosages of NaB and WA through MTT analysis. (**A**) MCF10A cells when treated with single and combined dosage concentrations of 2.5 mM NaB, 4 mM NaB, 0.4 mM NaB, 0.4 µM WA, and 0.2 µM WA, the noncancerous epithelial cell line did not display any significant toxicity when treated for 72 h. (**B**) MCF-7 cells treated with multiple concentrations of single and combined NaB and WA, showing the most significance with lower concentrations of 2.5 mM NaB and 0.2 µM WA. (**C**) Single and combined concentrations of WA and NaB showed the most significance with 2.5 mM NaB and 0.2 µM under MDA-MB-157 cell usage. (**D**) MDA-MB-231 treated with single and combined dosage concentrations of 2.5 mM and 0.2 µM, showing the most significance. An ordinary one-way ANOVA was conducted to compute *p*-values for MCF10A, MCF−7, MDA−MB−157, and MDA−MB−231 cells as 0.0855, <0.0001, <0.0001, and <0.0001, respectively. Values were represented as mean ± SEM (Standard Error of Mean) from three independent experiments (n = 3), each with six replicates per condition. Significant differences were reported as * (*p* < 0.05), ** (*p* < 0.01), and **** (*p* < 0.0001). Abbreviations: WA; withaferin A—steroidal lactone, NaB; sodium butyrate—short chain fatty acid, mM—millimolar, µM—micromolar.

Within this study, we first utilized different dosages of NaB and WA to test for the impact of cellular viability on triple-negative [ER (−) PR (−)] MDA−MB−157, MDA−MB−231 breast cancer cell lines, ER (+) MCF−7 breast cancer cell line, and the control MCF10A non-tumorigenic epithelial cell line utilizing a 3-(4,5-dimethylthiazol-2-yl)-2,5-diphenyltetrazolium bromide (MTT) assay. We have reported no toxicity to MCF10A at various dosages of 4 mM NaB, 2.5 mM NaB, 0.4 mM NaB, 0.4 µM WA, and 0.2 µM WA as seen in Figure 2.

SynergyFinder Plus utilizes a variety of functions such as sensitivity maps, synergy analysis, and combination effect visualization [20]. The synergy score ranges from 20 to −20 (synergistic to antagonistic capabilities) and can be displayed via 3 plots: Heatmap, contour plot, and interactive 3D surface. The Heatmap plots displaying synergy scores between NaB and WA for each cell line used can be found within the Supplementary Figures S1–S4. MCF10A cells had a synergy mean score of −7.19, and since some of the values are higher than 100%, the plots can be interpreted in multiple ways. As depicted in Figure S1 illustration, most of the calculated combination index (CI) values are within the negative range except for the combination of 2.5 mM NaB and 0.2 µM WA. The Heatmap Supplementary Figure S1 has a medial synergy score of 5.71 for the combination of 2.5 mM NaB and 0.2 WA. All other combinations have a medium antagonistic score of −2.79 to a low antagonist synergy score of −19.13. Since our results depicted MCF10A cells treated with 2.5 mM NaB and 0.2 µM WA having a measurably lower cell viability percentage compared to the other dosage combinations in Figure 2A, we treated MDA−MB−157, MDA—MB—231, and MCF—7 with the same dosages alone and in combination.

MCF−7 cells displayed a significant decrease in cell viability when treated with 2.5 mM NaB, 0.2 µM WA, and 0.4 µM WA for a single dose and combination within Figure 2B. Supplementary Figure S2 illustrate anatagonistic capabilities due to the mean synergy score of −35.59 and the only synergy plot score of 15.18 was found for the combination of 2.5 mM and 0.2 µM WA. MDA−MB−157 cells also depict a significant difference from the control and combination/single concentrations of 2.5 mM NaB, 0.4 µM WA, and 0.2 µM WA in Figure 2C. Supplementary Figure S3 illustrate the synergy score mean of −17.25 for MDA−MB−157 cells. The lowest synergy score of 15.35 on the Heatmap plot in Supplementary Figure S3 is within the 2.5 mM NaB and 0.2 µM WA combination. MDA—MB—231 has a synergy mean score of −4.09 with the combination of 2.5 mM and 0.2 µM WA score of 4.33 in Supplementary Figure S4.

**Figure 3:**
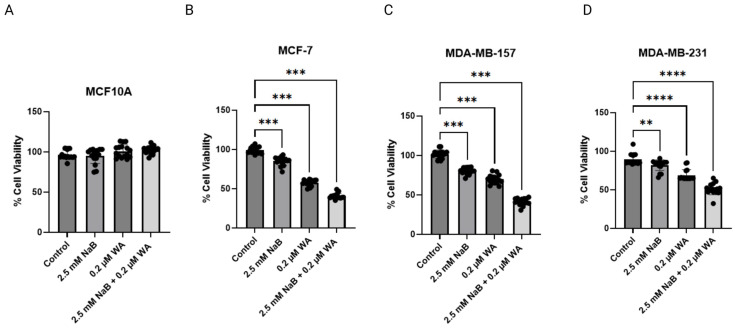
Inhibition of cellular viability for breast cancer cell lines with 2.5 mM NaB and 0.2 µM WA through Neutral red assay. (**A**) MCF10A, when treated with 2.5 mM NaB and 0.2 µM WA did not depict toxicity. While MCF−7 in (**B**), MDA−MB−157 in (**C**), and MDA-MB-231 in (**D**) displayed a significant decrease in cell viability when comparing the single dose/combination groups to the control group. The treatment was 72 h with dietary agents before conducting the Neutral Red assay. An ordinary one-way ANOVA was conducted to compute *p*-values for MCF10A, MCF−7, MDA−MB−157, and MDA-MB-231 as 0.012, <0.001, <0.001, and <0.0001, respectively. Values were represented as mean ± SEM (Standard Error of Mean) from three independent experiments (n = 3), each with four replicates per condition. Significant differences were reported as ** (*p* < 0.01), *** (*p* < 0.001), and **** (*p* < 0.0001). Abbreviations: WA; withaferin A—steroidal lactone, NaB; sodium butyrate—short chain fatty acid, mM—millimolar, µM—micromolar.

To ensure that the 2.5 mM NaB and 0.2 µM WA combination decreasing cell viability was consistent for MDA−MB−157, MDA-MB-231, and MCF−7 from the 3-(4,5-dimethylthiazol-2-yl)-2,5-diphenyltetrazolium bromide (MTT) assay, we conducted a Neutral red assay, and the outcome is illustrated in Figure 3.

A Neutral red assay can determine cell viability in an autophagy or an acidic microenvironment in vitro [21]. MCF10A displayed no toxicity when treated with 2.5 mM NaB and 0.2 µM WA as analyzed with the Neutral red assay in Figure 3A. MCF−7 did display a significant decrease in cell viability between single and combination 2.5 mM NaB/0.2 µM WA groups compared to the control in Figure 3B. MDA−MB−157 depicted a significant decrease in cell viability in Figure 3C when treated with 2.5 mM NaB and 0.2 µM WA alone and in combination, when compared to the control group, once analyzed with the Neutral red assay. MDA−MB−231 also depicted a significant decrease in cellular viability when treated with 2.5 mM NaB and 0.2 µM WA combinatorial group, with a significance value of 0.012.

### 3.2. Combinatorial NaB and WA Decreased DNMT’s Protein Expression

**Figure 4:**
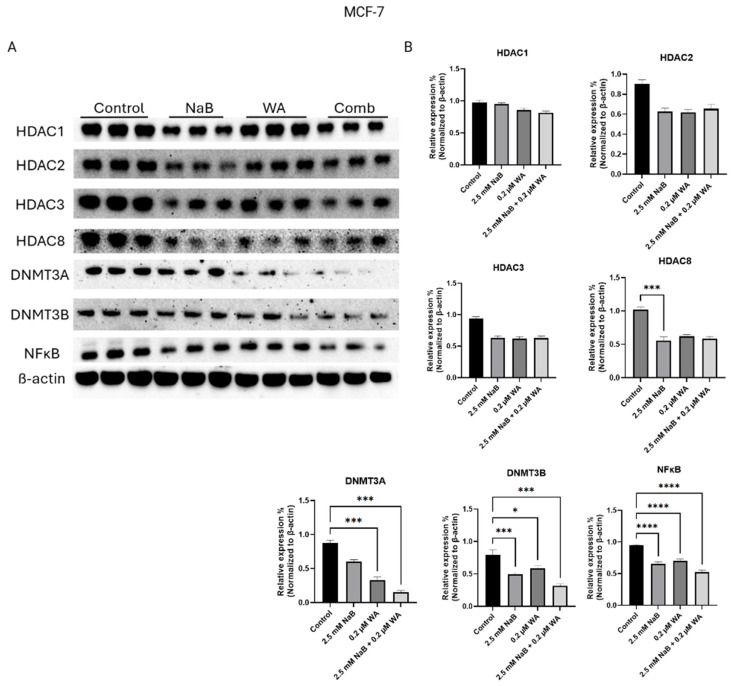
Protein expression decreased for MCF−7 cells when treated with NaB and WA for HDAC8, DNMT3A, and DNMT3B. (**A**) Depiction of the Western blot bands visualized utilizing ChemiDocTM Imaging Systems, and a clear decrease in protein expression is illustrated for DNMT3A. (**B**) Image J software harnessed to quantify protein bands normalized to β-actin. Values are represented as mean ± SEM (Standard Error of Mean) from three independent experiments (n = 3), each with three replicates per condition. Significant differences are reported as * (*p* < 0.05), *** (*p* < 0.001), **** (*p* < 0.0001). Abbreviations: Comb—combination, WA; withaferin A—steroidal lactone, NaB; sodium butyrate—short chain fatty acid, mM—millimolar, µM—micromolar, HDAC; histone deacetylase—enzyme, DNMT; DNA methyltransferase—enzyme, NF-κB—nuclear factor kappa-light-chain-enhancer of activated B-cells.

After gathering significance from the combination of 2.5 mM NaB and 0.2 µM WA treatment on MCF−7, MDA−MB−231, and MDA−MB−157 breast cancer cell lines, we next sought to analyze the ability of both compounds to decrease specific epigenetic targets. WA is a plant-derived steroidal lactone that is present in Ashwagandha (*Withania somnifera*) and observed to inhibit DNMT in breast cancer [15,22]. NaB is a short-chain fatty acid (SCFA) derived from the gut microbiota during fermentation of undigested carbohydrates [23]. NaB has become prevalent in recent studies as an HDAC inhibitor for various diseases through the induction of apoptosis [24]. Figure 4 illustrates the western blot bands and protein quantification for MCF−7 cells, highlighting targets Class 1 histones and de novo DNA methyltransferases. Figure 4B protein quantification of target DNMT3A has a *p*-value of <0.001, illustrating a decrease of expression for WA and the combinatorial group, also seen in Figure 4A within Row 5. DNMT3B also depicts a decrease in expression with a *p*-value of <0.001 as well. Figure 4A also depicts protein target HDAC8 having a reduction of expression with a *p*-value of <0.001 in Figure 4B.

**Figure 5:**
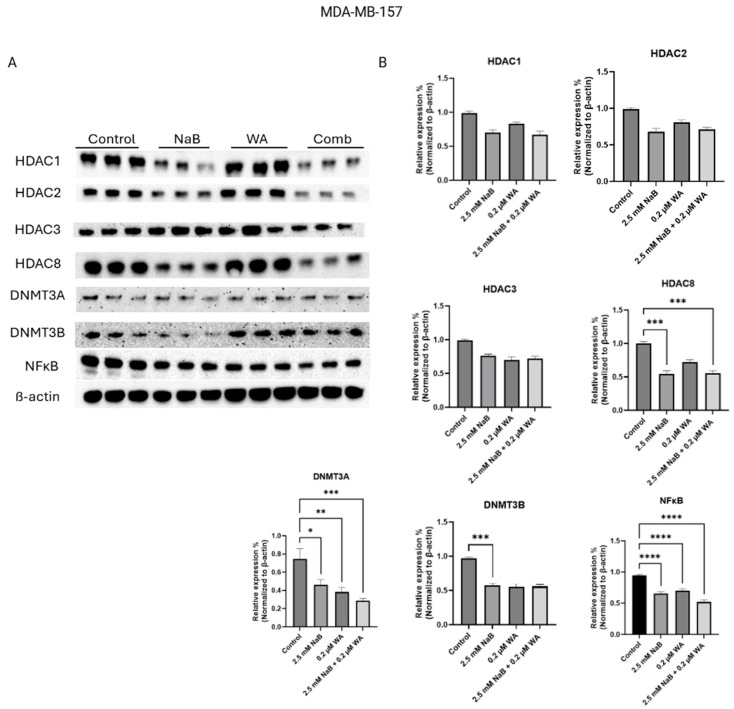
Protein expression decreased for MDA−MB−157 cells when treated with NaB for HDAC8, and multiple groups within DNMT3A, DNMT3B, and NF-κB targets. (**A**) Depiction of the Western blot bands visualized utilizing ChemiDocTM Imaging Systems and a clear decrease in protein expression is illustrated for DNMT3B. (**B**) Image J software harnessed to quantify protein bands normalized to β-actin. Values were represented as mean ± SEM (Standard Error of Mean) from three independent experiments (n = 3), each with three replicates per condition. Significant differences are reported as * (*p* < 0.05), ** (*p* < 0.01), *** (*p* < 0.001), and **** (*p* < 0.0001). Abbreviations: Comb—combination, WA; withaferin A—steroidal lactone, NaB; sodium butyrate—short chain fatty acid, mM—millimolar, µM—micromolar, HDAC; histone deacetylase—enzyme, DNMT; DNA methyltransferase—enzyme, NF-κB—nuclear factor kappa-light-chain-enhancer of activated B-cells.

Figure 5 illustrates the western blot bands and protein quantification for MDA−MB−157 cells, highlighting targets Class 1 histones and de novo DNA methyltransferases. Figure 5B protein quantification of target DNMT3B has a *p*-value of <0.001 for the NaB group, illustrating a decrease of protein expression also seen in Figure 5B within Row 6. HDAC8 and DNMT3A targets also have a significant decrease in protein expression with a *p*-value of <0.001. NF-κB protein expression decreased in all groups compared to the control with a *p*-value of <0.0001.

**Figure 6:**
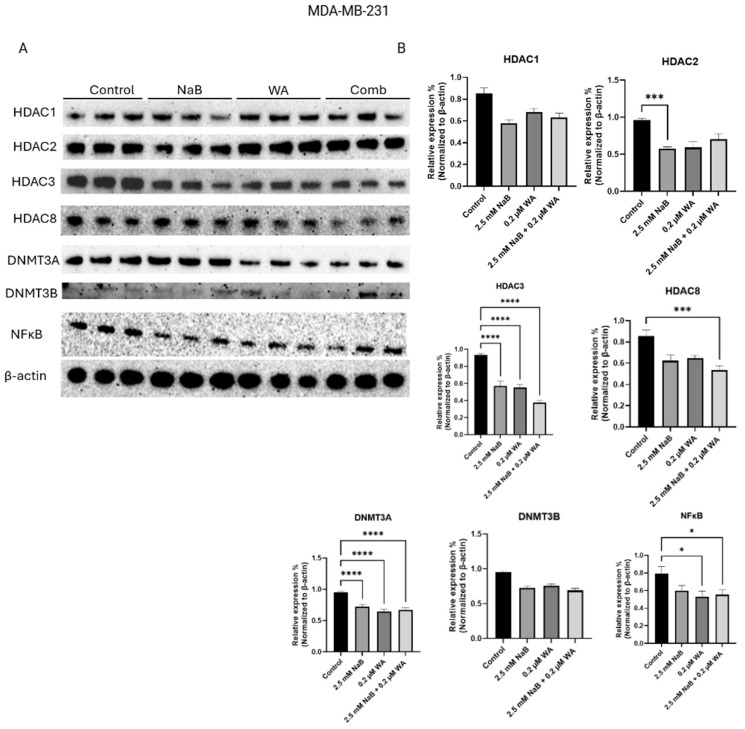
Protein expression decreased for MDA−MB−231 cells when treated with WA and NaB for HDAC3, HDAC8, and multiple groups within DNMT3A, and NF-κB targets. (**A**) Depiction of the Western blot bands visualized utilizing ChemiDocTM Imaging Systems and a clear decrease in protein expression is illustrated for DNMT3B, even though statistical significance is not seen graphically. (**B**) Image J software harnessed to quantify protein bands normalized to β-actin. Values were represented as mean ± SEM (Standard Error of Mean) from three independent experiments (n = 3), each with three replicates per condition. Significant differences are reported as * (*p* < 0.05), *** (*p* < 0.001), and **** (*p* < 0.0001). Abbreviations: Comb—combination, WA; withaferin A—steroidal lactone, NaB; sodium butyrate—short chain fatty acid, mM—millimolar, µM—micromolar, HDAC; histone deacetylase—enzyme, DNMT; DNA methyltransferase—enzyme, NF-κB—nuclear factor kappa-light-chain-enhancer of activated B-cells.

Figure 6 illustrates the western blot bands and protein quantification for MDA−MB−231 cells, highlighting targets Class 1 histones and de novo DNA methyltransferases. Figure 6B protein quantification of target HDAC2 as a *p*-value of 0.0001 for the NaB group, illustrating a decrease of protein expression also seen in Figure 6B within Row 3. HDAC3 *p*-value is <0.0001, HDAC8 *p*-value is 0.0003, DNMT3A *p*-value is <0.0001, and NF-κB *p*-value is 0.0320.

### 3.3. Combined Anti-Inflammatory Agents NaB and WA Inhibited NFκB1 Gene Expression

**Figure 7:**
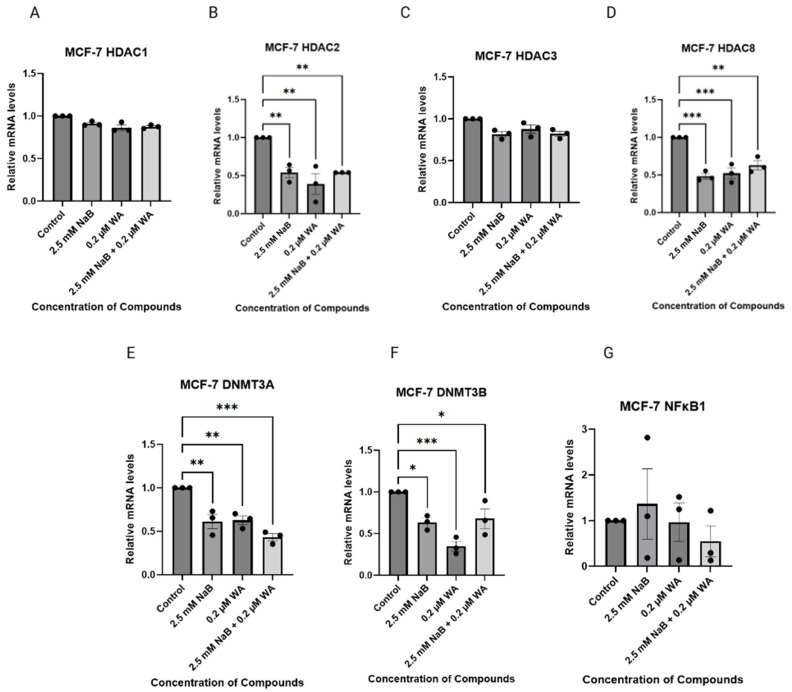
Combinatorial treatment with WA and NaB changes *DNMT* relative mRNA levels in MCF—7 cells. qRT-PCR was performed on WA and NaB treated MCF−7 cells. (**A**) depicts the relative mRNA levels of *HDAC1*. (**B**) depicts the relative mRNA levels of *HDAC2* with *p*-value of 0.0024. (**C**) depicts the relative mRNA levels of *HDAC3*. (**D**) depicts the relative mRNA levels of *HDAC8* with a *p*-value of 0.0003. (**E**) depicts the relative mRNA levels of *DNMT3A* with a *p*-value of 0.0004. (**F**) depicts the relative mRNA levels of *DNMT3B* with a *p*-value of 0.0014. (**G**) depicts the relative mRNA levels of *NFκB1*. Values were represented as mean ± SEM (Standard Error of Mean) from three independent experiments (n = 3), each with three replicates per condition. Significant differences were reported as * (*p* < 0.05), ** (*p* < 0.01), and *** (*p* < 0.001). Abbreviations: WA; withaferin A—steroidal lactone, NaB; sodium butyrate—short chain fatty acid, mM—millimolar, µM—micromolar, HDAC; histone deacetylase—enzyme, DNMT; DNA methyltransferase—enzyme, NF-κB—nuclear factor kappa-light-chain-enhancer of activated B-cells.

We have utilized Quantitative real-time PCR (qRT-PCR) to analyze the inhibitory gene expression capabilities of MCF−7, MDA−MB−231, and MDA−MB−157 cells when treated with 2.5 mM NaB and 0.2 µM WA. Figure 7 depicts the various targets of Class I HDACs, de novo DNA methyltransferases, and *NFκB1* progression genes for MCF—7 cells. Figure 7B depicts a decrease in *HDAC2* relative gene expression with a *p*-value of 0.0024. Gene targets *HDAC8*, *DNMT3A*, and *DNMT3B* illustrated a significant decrease in gene expression with *p*-values of 0.0003, 0.0004, and 0.0014.

**Figure 8:**
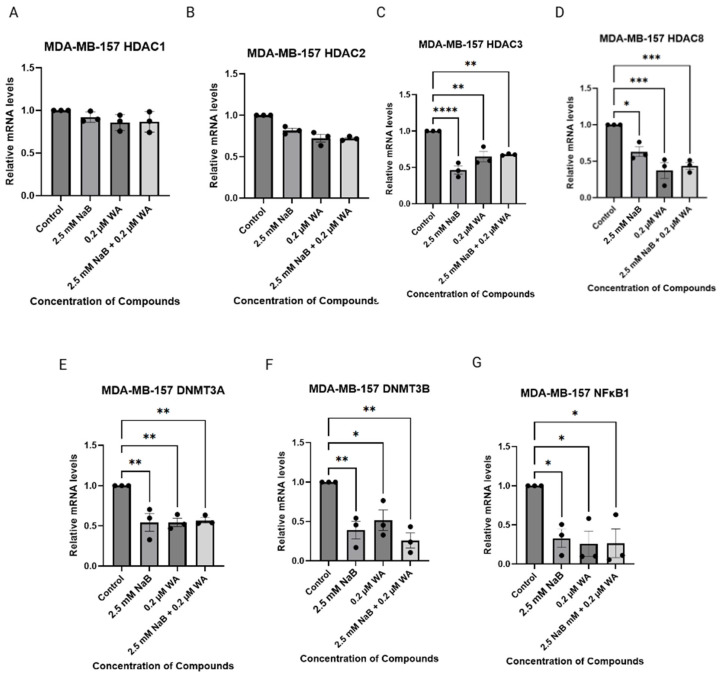
qRT-PCR was performed on WA and NaB treated MDA−MB−157 cells. (**A**) depicts the relative mRNA levels of *HDAC1.* (**B**) depicts the relative mRNA levels of *HDAC2.* (**C**) depicts the relative mRNA levels of *HDAC3* with a *p*-value of 0.0002. (**D**) depicts the relative mRNA levels of *HDAC8* with a *p*-value of 0.0007. (**E**) depicts the relative mRNA levels of *DNMT3A* with a *p*-value of 0.0022. (**F**) depicts the relative mRNA levels of *DNMT3B* with a *p*-value of 0.0034. (**G**) depicts the relative mRNA levels of *NFκB1* with a *p*-value of 0.0123. Significant differences were reported as * (*p* < 0.05), ** (*p* < 0.01), *** (*p* < 0.001), and **** (*p* < 0.0001). Values were represented as mean ± SEM (Standard Error of Mean) from three independent experiments (n = 3), each with three replicates per condition. Abbreviations: WA; withaferin A—steroidal lactone, NaB; sodium butyrate—short chain fatty acid, mM—millimolar, µM—micromolar, HDAC; histone deacetylase—enzyme, DNMT; DNA methyltransferase—enzyme, NF-κB—nuclear factor kappa-light-chain-enhancer of activated B-cells.

MDA−MB−157 cells were also treated with 2.5 mM of NaB and 0.2 µM of WA for 72 h before conducting qRT-PCR on the various targets of Class I HDACs, de novo DNA methyltransferases, and *NFκB1* gene. The association of *NFκB1* was needed to ensure the relation of HDACs with the inflammation-driven pathway [25]. Significance was identified during analysis as illustrated in Figure 8, within *HDAC3*, *HDAC8*, *DNMT3A*, *DNMT3B*, and *NFκB1*.

**Figure 9:**
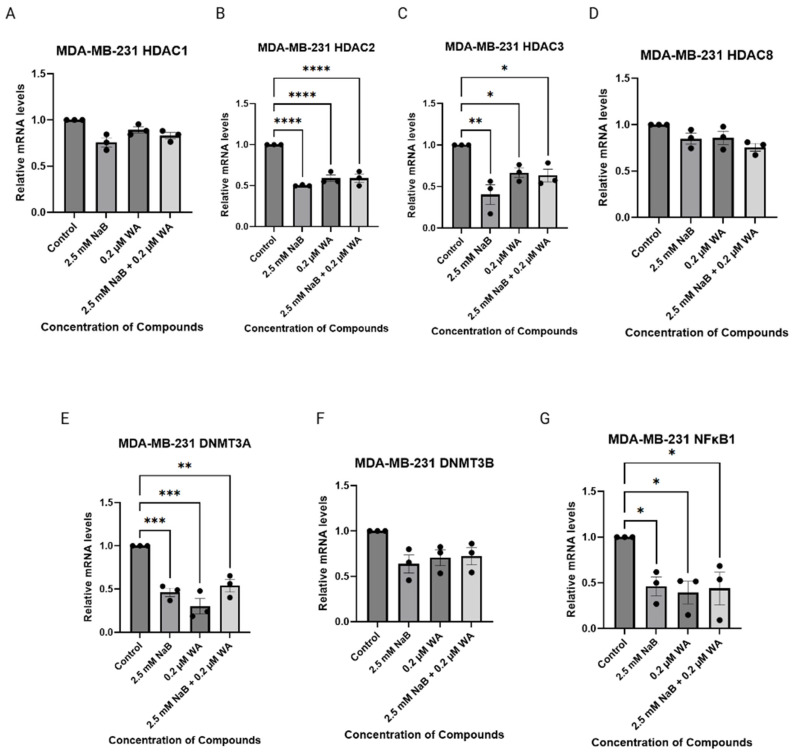
qRT-PCR was performed on WA and NaB treated MDA−MB−231 cells. (**A**) depicts the relative mRNA levels of *HDAC1.* (**B**) depicts the relative mRNA levels of *HDAC2* with a *p*-value of <0.0001. (**C**) depicts the relative mRNA levels of *HDAC3* with a *p*-value of 0.0039. (**D**) depicts the relative mRNA levels of *HDAC8.* (**E**) depicts the relative mRNA levels of *DNMT3A* with a *p*-value of 0.0003. (**F**) depicts the relative mRNA levels of *DNMT3B.* (**G**) depicts the relative mRNA levels of *NFκB1* with a *p*-value of 0.0219. Significant differences were reported as * (*p* < 0.05), ** (*p* < 0.01), *** (*p* < 0.001), and **** (*p* < 0.0001). Values were represented as mean ± SEM (Standard Error of Mean) from three independent experiments (n = 3), each with four replicates per condition. Abbreviations: WA; withaferin A—steroidal lactone, NaB; sodium butyrate—short chain fatty acid, mM—millimolar, µM—micromolar, HDAC; histone deacetylase—enzyme, DNMT; DNA methyltransferase—enzyme, NF-κB—nuclear factor kappa-light-chain-enhancer of activated B-cells.

MDA−MB−231 cells were treated with 2.5 mM of NaB and 0.2 µM of WA for 72 h before conducting qRT-PCR on the various targets of Class I HDACs, de novo DNA methyltransferases, and *NFκB1* gene. Significance was identified during analysis as illustrated in Figure 9, within *HDAC2*, *HDAC3*, *DNMT3A*, and *NFκB1*.

### 3.4. Enzymatic Activity Changes in MCF−7, MDA-MB-231, and MDA−MB−157 Cells When Treated with NaB and WA

**Figure 10:**
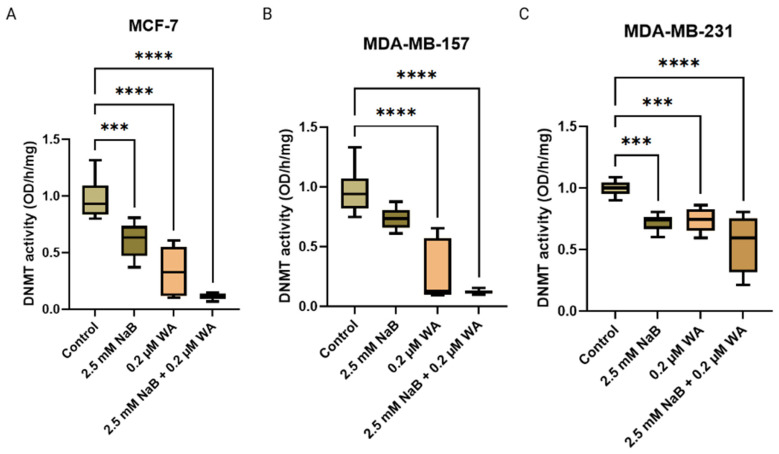
Enzymatic activity for MCF−7, MDA−MB−157, and MDA—MB—231. DNMT activity for (**A**) MCF−7 cells with *p*-value of <0.0001, (**B**) MDA−MB−157 cells with *p*-value of <0.0001, and (**C**) MDA—MB—231 cells with a *p*-value of <0.0001 when treated with 2.5 mM NaB and 0.2 µM WA. Values were represented as mean ± SEM from one independent experiment (n = 3), each with six replicates per condition. Significant differences were reported as *** (*p* < 0.001) and **** (*p* < 0.0001). Abbreviations: WA; withaferin A—steroidal lactone, NaB; sodium butyrate—short chain fatty acid, mM—millimolar, µM—micromolar, DNMT; DNA methyltransferase—enzyme.

Since our Western blot analysis, illustrated in Figure 4, Figure 5 and Figure 6, depicts a significant decrease in DNMT3A and DNMT3B protein expression for all breast cancer cell lines utilized, respectively, we next sought to validate these findings with the Epigentek DNMT Activity/Inhibition ELISA Easy Kit. We found a significant decrease in DNMT activity for 2.5 mM of NaB and 0.2 M of WA single/combination groups compared to the control, illustrated in Figure 10.

## 4. Discussion

Triple-negative breast cancer (TNBC) grows in the body and spreads quickly compared to other subtypes, and it is more common in women 40 years of age or younger compared to other subtypes. Some may ask why this is the case and how these statistics can be better understood. By utilizing the TNBC cell lines MDA−MB−157, MDA−MB−231, and an ER (+) cell line MCF−7, we sought to elucidate how these diverse breast cancer cell lines would respond to treatment with the short-chain fatty acid sodium butyrate (NaB) and the active component within Ashwagandha, withaferin A, to evaluate changes in breast cancer progression. Could the addition of certain dietary agents combined with modern treatments, such as radiation therapy, chemotherapy, or hormonal therapy, be sufficient to decrease the chance of death for black women regarding breast cancer?

Our previous studies have shown that different phytochemicals such as sulforaphane (SFN), genistein, epigallocatechin−3−gallate (EGCG), and grape seed proanthocyanidins (GSPs) have been beneficial in decreasing the progression of breast cancer in vivo and in vitro. SFN is an active component found in most cruciferous vegetables, such as broccoli sprouts (BSp) that has displayed histone deacetylase (HDAC) inhibition capabilities through increasing the rate of apoptosis in MCF−7 and MDA−MB−231 cells, along with reducing tumor volume/size in a C3(1)-SV40 Tag transgenic mouse model (SV40) [26]. Genistein (GE) is a natural isoflavone found in soybean products that has a role as a DNA methyltransferase (DNMT) inhibitor and has suppressed breast tumorigenesis by targeting tumor suppressor genes *p21WAF1*(*p21*) and *p16INK4a*(*p16*) [27]. EGCG is a catechin found in green tea with DNMT inhibition capabilities that has been shown to prevent breast cancer through inhibition of tumor angiogenesis, along with working synergistically with SFN-rich broccoli sprouts to reduce tumor volume over time [28]. EGCG-oriented green tea polyphenols have been shown in studies to be potent antioxidants and have worked with genistein, withaferin A, curcumin, resveratrol, and guggulsterone to modify breast cancer cell lines through DNA methylation [29].

Our current study of NaB and WA’s impact on breast cancer in vitro is novel and has not been analyzed to our knowledge. The anti-inflammatory agents utilized can have major impacts on breast cancer through various ways, including altering the tumor microenvironment, causing a change in epigenetic regulatory mechanisms, and decreasing breast cancer progression through inhibiting tumor suppressor gene expression [30,31,32]. NaB has been used in combination with EGCG to induce cell cycle arrest in colon cells and promote apoptosis [33]. We have established a decrease in cell viability utilizing various dosages of NaB, especially 2.5 mM, as seen in Figure 2B,C for MCF−7 and MDA−MB−157 cells. Our study indicates that the dosage of 2.5 mM NaB and 0.2 µM WA was optimal in efficiency for decreasing cell viability in the TNBC and ER (+) cell lines. The dosage compensation was the most synergistic for breast cancer cell lines MDA-MB-157 and MCF-7 and had additive affects for MDA-MB-231 and the control cell line MCF10A at the 2.5 mM NaB and 0.2 µM WA dosage. Further investigation utilizing the neutral red assay revealed a significant decrease in each NaB and WA group alone/combined compared to the control group as shown in Figure 3B,C. Analysis of the Neutral red assay relies on the ability of viable cells to uptake the red dye within the lysosomes of the cell and is ideal for evaluating the efficacy of anticancer drugs in distinguishing tumor microenvironments [21]. Studies have shown that WA has even demonstrated macrophage clearance capabilities when used to treat *S. aureus* in zebrafish with the Neutral red assay [34]. The concentration of 0.2 µM WA in our study has exhibited a decline of DNMT3A protein expression for MCF−7 cells with a *p*-value of <0.001, according to Figure 4B. These findings are aligned with the previous discovery for WA combined with SFN to increase global methylation in MCF−7 and MDA−MB−231 cells [15]. Figure 5B also depicts a decrease in DNMT3B protein expression for MDA−MB−157 cells.

NaB and WA have specific mechanisms within the NF-κB pathway that include directing multiple components involved in activation. NaB functions as a histone inhibitor that targets the p65 subunit, reducing portions of the pathway from relocating to the nucleus and activating survival genes. This, in turn, reduces the ability of phosphorylation and degradation of IκBα (an inhibitor that keeps NF-κB within the cytoplasm) [35]. WA also inhibits IKKβ kinase (IKK), which again immobilizes NF-κB from leaving the cytoplasm and being transported to the nucleus for transcription of cell survival genes [36]. Our analysis depicts a significant decline in *DNMT3B* gene expression for MCF−7 cells when treated with 2.5 mM NaB and 0.2 µM WA as shown in Figure 7F (*p*-value 0.0014). These findings indicate that the ER (+) cell line can be impacted by the anti-inflammatory combination within our study. Additionally, in Figure 9F, not producing the same decline in DNMT3B for the TNBC cell line may be due to the lack of those ER, PR, and HER2 receptors and reliance on alternative survival pathways such as NF-κB, Epidermal Growth Factor Receptor (EGFR), and Phosphatidylinositol 3-kinase/Akt (PI3K/AKT) [30,37]. Also, since the post-transcriptional regulation or protein stability could play a role in different results for the targets, the observed phenotypic effects are likely due to the cumulative impact on both gene and protein networks.

We also wanted to validate the change in DNMT protein expression analyzed from Western blotting in Figure 4, Figure 5 and Figure 6 by utilizing the Epigentek DNMT Activity/Inhibition ELISA Easy Kit. In Figure 10, there is a significant decline in DNMT activity for NaB and WA in combination/alone, with a *p*-value of <0.0001. This study illustrates that NaB and WA have additive capabilities in decreasing cell viability in MCF−7, MDA−MB−231, and MDA−MB−157 cell lines. There is also a diverse response to these agents depending on the availability of hormonal receptors. Additional studies could be done involving spheroid formation to have a more parallel representation of the tumor microenvironment with the human body. Investigators have utilized spheroid formation to analyze inhibitory impacts of phytochemicals on breast cancer progression [38]. Additionally, the translation of the efficacy of these agents will need to be performed in vivo in future studies to increase translational relevance. The ER (+) and TNBC cell lines were utilized to compare the impact of a hormonal molecular subtype derived from a white female patient and a molecular subtype derived from a black female patient. Testing these agents with additional breast cancer cell lines and conducting in vivo studies would strengthen the possible clinical applications of our findings. Additionally, the ability of these agents to reduce inflammation could be investigated by analyzing the capability of reducing IκB kinase and MAPKs pathway [39]. By analyzing the ability of the anti-inflammatory agents to block the MAPKs pathway, we could elucidate the mechanisms involving that aspect in future studies.

NaB and WA have also been illustrated as natural compounds that could help modulate the anti-inflammatory effects needed to reduce symptoms from irritable bowel syndrome (IBS). NaB, being a short-chain fatty acid produced by gut microbiota, can modulate the intestinal microbiota that is often off balance in IBS. WA is also a great candidate due to the anti-inflammatory and epigenetic effects that will need more exploration to derive the exact points of usage for IBS modulation. The connection between the NF-κB pathway and IBS has been highlighted in previous studies that demonstrated inhibition of the NF-κB pathway could treat side effects of IBS, such as diarrhea [40]. Sodium butyrate, utilized in a recent study, found the SCFA ability to alleviate additional symptoms of irritable bowel disease (IBD) in mice that reduced the disease activity index that incorporates the percent weight loss, stool, and bleeding amount [41]. This relates to the many side effects that could be treated by the compounds as adjuvant strategies in breast cancer treatment. Of course, there are limitations of this study that include the use of an in vitro model and a lack of causality due to the focus on the combinatorial effects of the compounds in vitro. Studies have shown that phytochemicals utilized can mitigate chemoresistance in pathways such as NF-κB, and there are emerging drivers that can impact the progression of breast cancer once overcoming the delicate unknowns that persist within the field [42]. These unknowns include the concept of combining Fecal Microbiota transplantation (FMT), probiotics, and dietary interventions to inhibit breast cancer progression and/or side effects from treatment [43]. The study we have conducted resolved the question of sodium butyrate and withaferin A impact on breast cancer cell lines, and we showed that this combination illustrated an additive or synergistic relationship at the 2.5 mM NaB and 0.2 µM WA dosage, depending on cell type. There would need to be additional investigation to increase the amount to ensure translational usage for human consumption after being tested in an in vivo model.

There has been work done that correlates with findings from others in the field, indicating that withaferin A is able to downregulate and block NF-κB due to the generated reactive oxygen species (ROS) produced when targeting renal cell carcinoma for the tumor necrosis factor-related apoptosis-inducing ligand (TRAIL) drug [44]. Based on previous studies, we understand that withaferin A and sodium butyrate can be powerful compounds to utilize against breast cancer progression. The literature depicts this, and our study provides additional findings that are at the beginning of revealing additional capabilities of these compounds in a combinatorial environment. Both compounds have anti-inflammatory capabilities and were chosen strategically to identify the impact on the NF-κB pathway based on previous findings that include capabilities the compounds are known for, such as HDAC and DNMT inhibition [45,46]. Our studies have been successful at analyzing the impact of NaB and WA treatment on decreasing MCF−7, MDA−MB−231, and MDA−MB−157 breast cancer progression through a decline of cellular viability and DNMT expression.

## 5. Conclusions

Through cell viability analysis, protein expression, gene expression, and enzymatic activity assays, we were able to identify the role sodium butyrate and withaferin A have toward breast cancer cell line prevention. There are more quantitative methods that can be utilized to increase the causality between the compounds and the NF-κB pathway reduction to influence breast cancer inhibition. Future investigations are needed to elucidate the underlying mechanisms, particularly through functional analyses of NF-κB signaling, such as nuclear translocation and transcriptional activity assays, as well as through genetic or pharmacological modulation approaches. In addition, future work should explore key cellular processes, including apoptosis, cell cycle regulation, and oxidative stress, to better define the biological effects of the combined treatment. Finally, studies addressing clinically relevant dosing and the potential combination of these compounds with standard therapies may help clarify their applicability as adjuvant strategies in breast cancer treatment. The study is limited by the lack of mechanistic validation, as conclusions regarding NF-κB modulation are based primarily on expression data rather than more functional assays, which weakens casual interpretation. However, the findings did depict a decrease in cell viability due to an additive and synergistic relationship between NaB and WA within the indicated breast cancer cell lines utilized. There was also a decrease in protein expression for de novo DNA methyltransferases utilized and DNMT enzymatic activity. Lastly, we were able to identify a decrease within the protein and gene expression of NF-κB when treated with 2.5 mM NaB and 0.2 µM WA.

## Data Availability

The original contributions presented in this study are included in the article. Further inquiries can be directed to the corresponding author.

## Supplementary Materials

The following supporting information can be downloaded at: https://www.mdpi.com/article/10.3390/nu18061015/s1, Figure S1: MCF10A Heatmap mean Synergy score of −7.19 from SynergyFinder Plus; Figure S2: MCF-7 mean synergy score of −35.59 for Heatmap expressed through SynergyFinder Plus; Figure S3: MDA-MB-157 Heatmap with synergy mean score of −17.25 illustrated by SynergyFinder Plus; Figure S4: MDA-MB-231 Heatmap with synergy mean score of −4.09 illustrated by SynergyFinder Plus; Table S1: Information of primary antibodies for western blot in this study; Table S2: Primer sequences for real-time PCR analysis.
