# Supplementary material for: Combined Effects of Withaferin A and Sodium Butyrate on NF-κB Signaling and Epigenetic Regulation in Breast Cancer Cells
Source: Nutrients. 2026 Mar 23;18(6):1015. doi: 10.3390/nu18061015 (PMC13029483; doi:10.3390/nu18061015)
Supplement: Supplementary file 1 [file nutrients-18-01015-s001.zip › Table S1.pdf]

Supplementary Table S1. Information of primary antibodies for western blot in this study.

Antibody	MW/ kDa	Species	Source	Catalogue no.	Dilution ratio
Dnmt3a	130	Rabbit	Cell Signaling	3598S	1:1000
Dnmt3b	96	Rabbit	Cell Signaling	67259S	1:1000
Hdac1	62	Rabbit	Cell Signaling	34589S	1:1000
Hdac2	60	Rabbit	Cell Signaling	57156S	1:1000
Hdac3	49	Rabbit	Cell Signaling	85057S	1:1000
Hdac8	45	Rabbit	Cell Signaling	66042S	1:1000
NF-kappaB p65	65	Rabbit	Cell Signaling	8242S	1:1000
