# Supplementary material for: Combined Effects of Withaferin A and Sodium Butyrate on NF-κB Signaling and Epigenetic Regulation in Breast Cancer Cells
Source: Nutrients. 2026 Mar 23;18(6):1015. doi: 10.3390/nu18061015 (PMC13029483; doi:10.3390/nu18061015)
Supplement: Supplementary file 1 [file nutrients-18-01015-s001.zip › Table S2.pdf]

Supplementary Table S2. Primer sequences for real-time PCR analysis

Gene	Forward Primer Sequence (5′-3′)	Reverse Primer Sequence (5′-3′)
DNMT3A	GAAGTATCAGCACCCAGAAGAG	GGAGATGATGTCCAACCCTTT
DNMT3B	GGAGCCACGACGTAACAAATA	GTAAACTCTAGGCATCCGTCATC
HDAC1	CCAGTATTCGATGGCCTGTT	GATGTCCGTCTGCTTAT
HDAC2	CAGGTCCAGGAGTCAGAATAAC	CTACAAGGCCAGAGGGTTAAG
HDAC3	CAGGGACTTCAGCCTACTTTC	GAGGTAAGCCAGAGGCAATTA
HDAC8	ACTGCATAAGCAGATGAGGATAG	TTCTGGAGATGCAGATAAG
NFκB1	GGAAATTTGCGTGTGGAGTATTT	GTTGTAGTGGATGGTGGTACAG
GAPDH	CTCCTCACAGTTGCCATGTA	GTTGAGCACAGGGTACTTTATTG
