# Supplementary material for: Combined Effects of Withaferin A and Sodium Butyrate on NF-κB Signaling and Epigenetic Regulation in Breast Cancer Cells
Source: Nutrients. 2026 Mar 23;18(6):1015. doi: 10.3390/nu18061015 (PMC13029483; doi:10.3390/nu18061015)
Supplement: Supplementary file 1 [file nutrients-18-01015-s001.zip › Supplementary Information_CI Values.pdf]

Supplementary Information CI Value Plots

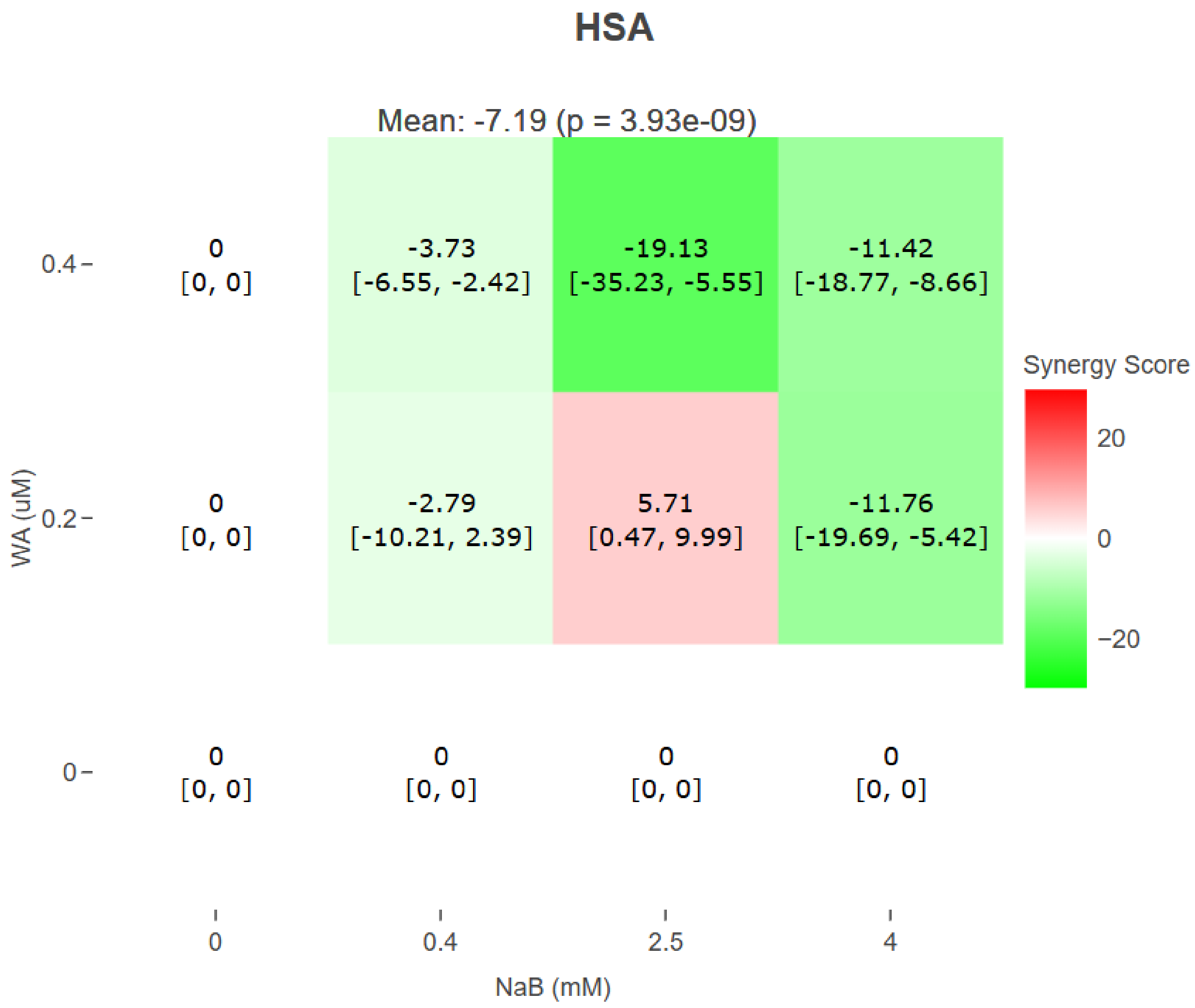

Figure S1: MCF10A Heatmap mean Synergy score of -7.19 from SynergyFinder Plus

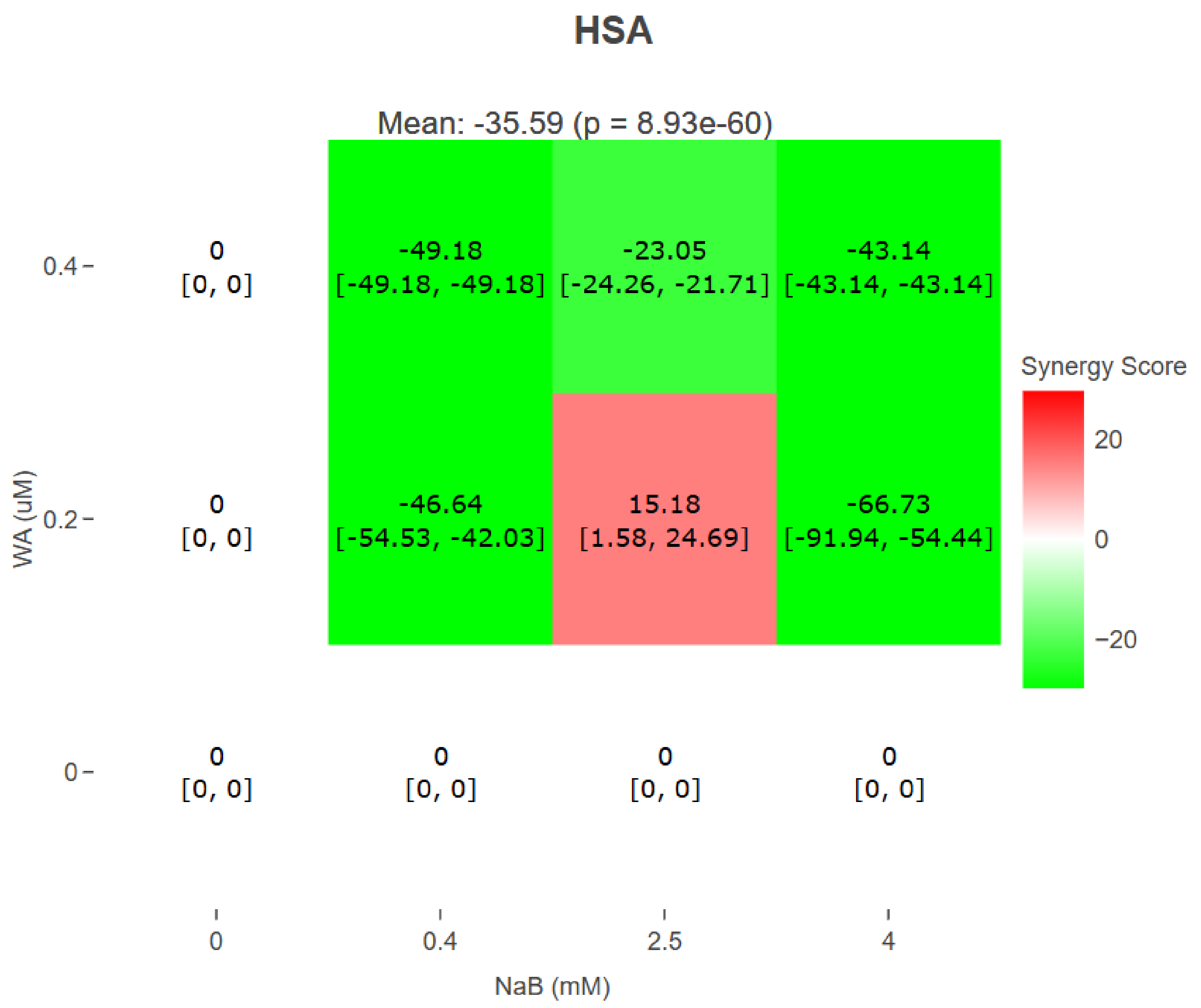

Figure S2: MCF-7 mean synergy score of -35.59 for Heatmap expressed through SynergyFinder Plus

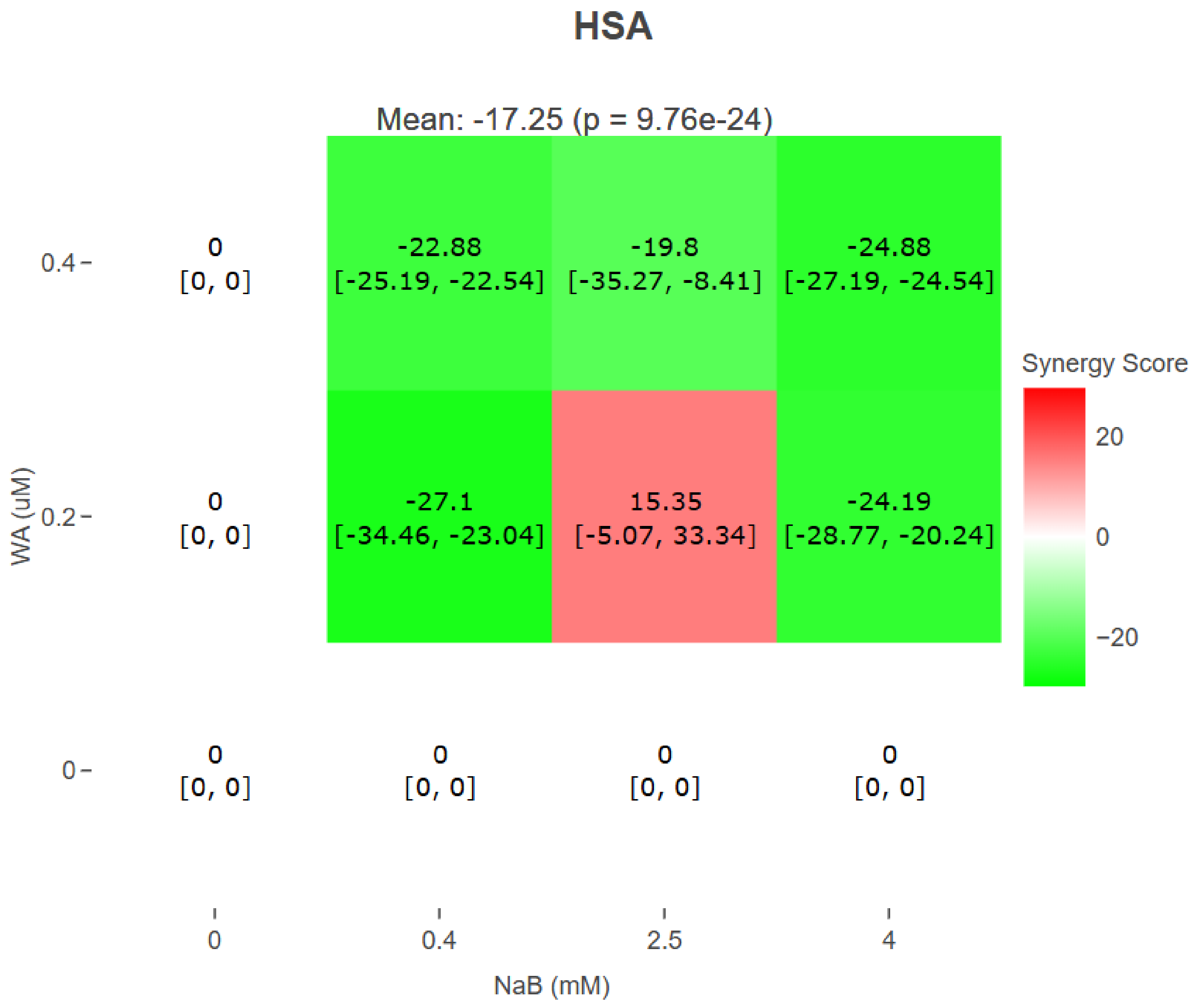

Figure S3: MDA-MB-157 Heatmap with synergy mean score of -17.25 illustrated by SynergyFinder Plus

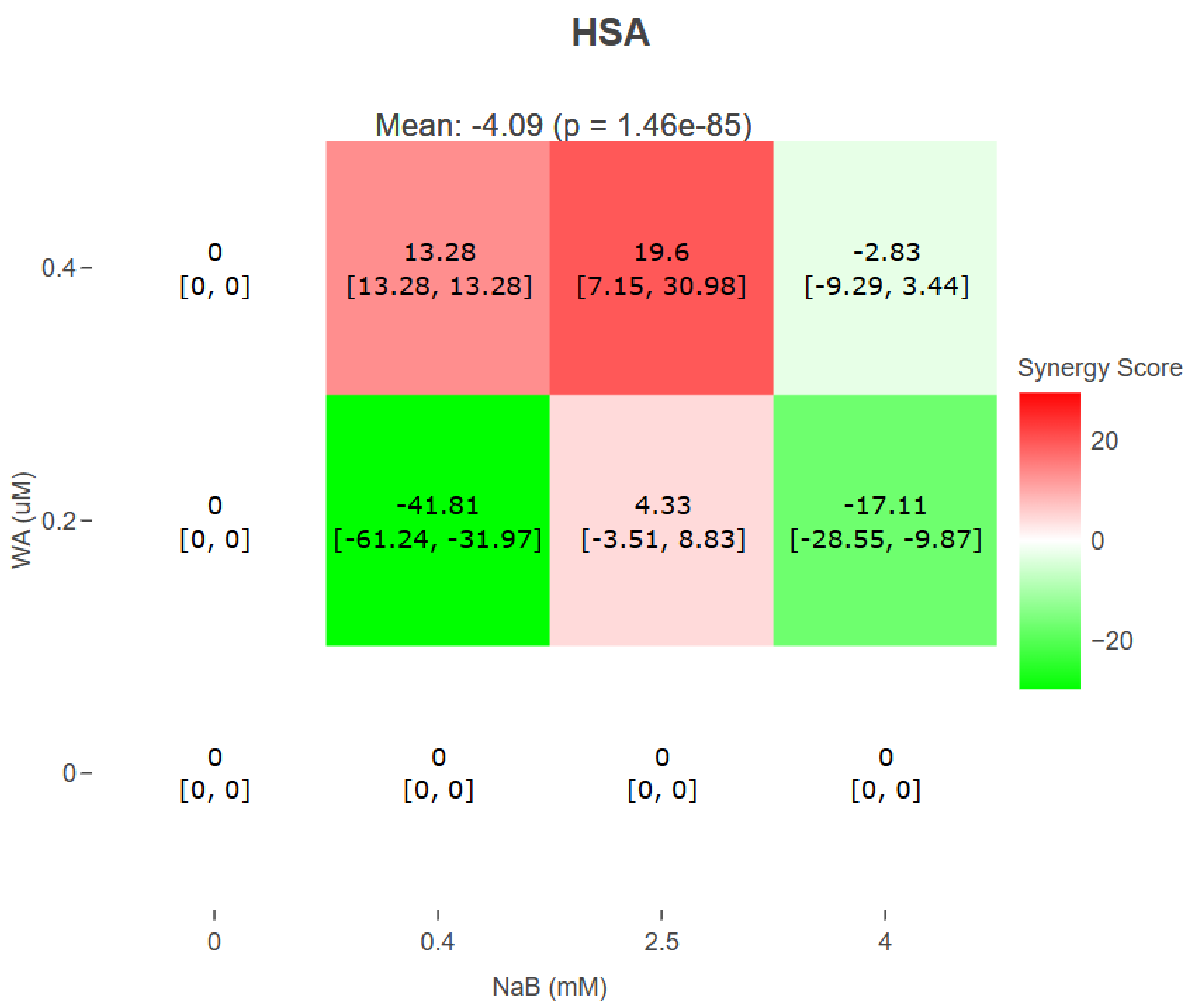

Figure S4: MDA-MB-231 Heatmap with synergy mean score of -4.09 illustrated by SynergyFinder Plus
